# EXPRESSION OF THE TARDIGRADE DAMAGE SUPPRESSOR PROTEIN IN THE YEAST SACCHAROMYCES CEREVISIAE

**DOI:** 10.1093/geroni/igad104.2489

**Published:** 2023-12-21

**Authors:** Wei-Hsuan Su, Jessica Smith, Evien Cheng, Samuel Schriner

**Affiliations:** University of California Irvine, Irvine, California, United States; UCI, Irvine, California, United States; UCI, Irvine, California, United States; UCI, Irvine, California, United States

## Abstract

Tardigrades are resilient organisms that can tolerate extremes in temperature, pH, pressure, and salinity. They are also exceptionally resistant to starvation, dehydration, and irradiation. The damage suppressor (Dsup) protein is thought to be unique to tardigrades and may act by coating and protecting nuclear DNA, particularly the nucleosomes. Nuclear DNA damage and mutation may be one of the driving forces of the aging process. In addition, there has been demonstrated a clear relationship between stress tolerance and aging. In this study, we are asking whether Dsup, when expressed in the yeast Saccharomyces cerevisiae, can afford yeast with an enhanced tolerance to environmental stresses, lowered spontaneous and induced mutation rates, and extended chronological lifespan. Yeast were constructed expressing Dsup with the strong promoter TEF on a high copy plasmid containing the 2 micron origin of replication. Preliminary results show that Dsup is able to enhance yeast survival against hydrogen peroxide, but surprisingly appeared to compromise survival to the DNA damaging agent ethyl methanesulfonate (EMS). Excessive expression of Dsup may explain the unexpected toxicity to EMS. We are therefore planning to test the expression of Dsup with a lower copy plasmid. We then plan to challenge the Dsup-expressing yeast to a variety of stresses, including extremes in temperature, high salt, and heavy metals. Induced mutant frequencies and spontaneous mutations rates at the CAN1 locus will be evaluated. Ultimately, we intend to determine whether Dsup can extend yeast chronological lifespan as a direct test of nuclear DNA damage in the regulation of lifespan.

